# Are Large‐Scale Differences in Temperature and Reindeer Management Regime Affecting the Quality of Reindeer's Summer Forage?

**DOI:** 10.1002/ece3.72500

**Published:** 2025-11-15

**Authors:** Fanny Berthelot, Audun Stien, Eeva M. Soininen, Torkild Tveraa, Hanna Böhner, Kari Anne Bråthen

**Affiliations:** ^1^ Department of Arctic and Marine Biology Arctic University of Norway (UiT) Tromsø Norway; ^2^ Norwegian Institute for Nature Research (NINA) Tromsø Norway

**Keywords:** defense compounds, food quality, foodscape, long‐term effects, near infrared spectroscopy, nutrients, plant chemical composition, plant functional group, reindeer herbivory

## Abstract

The chemical balance between essential nutrients and defense compounds in plants determines the quality of the forage available to herbivores and can be modified by both environmental conditions and herbivores themselves. We investigated whether climate and herbivory affect nutrient and defense compound concentrations across plant functional groups. Concentrations of nutrients—nitrogen (N) and phosphorus (P)—and defense compounds—silicon (Si) and phenolics (Ph)—were measured in plant samples from the locally most abundant species, collected in northern Norway across a gradient in summer temperature and different reindeer grazing regimes. Nutrient and defense compound concentrations varied substantially across species and plant functional groups. In addition, nutrients (N, P) declined over the summer, while defense compounds (Si, Ph) accumulated. Sites with a warmer climate had a stronger decrease in nutrient concentrations over the season. We found no evidence that long‐term contrasts in reindeer herbivory intensity affected the average nutritional quality within plant species. Overall, our results suggest that spatial variation in the nutritional quality in Arctic tundra vegetation is mainly determined by the species composition and by consistent changes over the summer season. In comparison, reindeer herbivory and local climate seem to have relatively little impact on the average nutrient and defense compound concentrations of the plant species, suggesting they mainly alter forage quality through effects on the functional and taxonomic composition of the vegetation.

## Graphical abstract

We evaluate whether temperature and reindeer herbivory lead to detectable patterns of variation in the quality of plant species at a large spatial scale, with results on both nutrients and defense compounds. We found that such effects are small, and thus suggest that the main determinants of the quality of the vegetation available to herbivore are the species composition of the pasture and the seasonal change in nutrient and defense compound concentrations towards the end of the growing season.

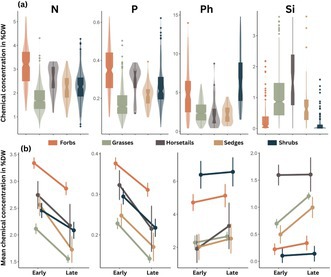

## 1 Introduction

Chemical balance between essential nutrients and defense compounds in plants determines the quality of the forage available to herbivores. The concentration of these compounds has previously been shown to vary substantially both between and within plant functional groups, plant species, tissues, ages and seasons (Mattson 1980; Kemppinen and Niittynen 2022; Thomas et al. 2019) and can be influenced by environmental factors such as temperature and moisture (Nybakken et al. 2008; Yue et al. 2019). Plant chemical composition can also be modified by the herbivores themselves by inducing a defense response in the plants (e.g., Massey et al. 2007), changing the plants' allocation of resources (Bryant and Reichardt 1992), making nutrients easily available through the fertilization of the soil (Barbero‐Palacios et al. 2023; Van der Waal et al. 2011), and by altering the taxonomic and functional composition of the plant community (Bardgett and Wardle 2003; Tuomi et al. 2019). Overall, these patterns and processes result in considerable spatial and temporal variation in herbivore forage quality, which in turn modifies herbivore habitat selection (Frye et al. 2013), foraging strategies (Bedoya‐Pérez et al. 2014), risk taking (Mella et al. 2015), and reproductive success (DeGabriel et al. 2009).

In the Arctic tundra, the increase in temperatures caused by global warming modifies vegetation. Warming has been reported to consistently increase tundra plant growth, especially in shrubs and graminoids (Elmendorf et al. 2012; Walker et al. 2006). The effect of warming on the chemical composition of Arctic tundra plants is, however, less clear. Experimental studies have found that increasing temperatures lead to a decline in average nutrient concentrations, an effect that may be mitigated by soil moisture (Petit Bon et al. 2023), while others report that the main effect of higher summer temperatures is a faster seasonal decline in nutrient concentrations (Doiron et al. 2014). Experimental studies of phenolic defense compounds have shown that plant phenol concentrations may decrease (Zhou et al. 2022), increase (Nybakken et al. 2008) or remain stable (Nybakken et al. 2011) in response to increased temperatures. We expand on previous work by evaluating whether these patterns are present across large‐scale spatial gradients in summer temperature and reindeer herbivory.

Reindeer ( *Rangifer tarandus* ) have a circumpolar distribution and are the most abundant large herbivore in the Arctic tundra. Although their impact is highly context dependent (Bernes et al. 2015), reindeer have been shown to modify the plant species composition (Bråthen et al. 2017; Bråthen and Oksanen 2001; Sundqvist et al. 2019) as well as the chemical composition of the plants through grazing (Petit Bon, Gunnarsdotter Inga, et al. 2020). In Northern Norway, reindeer are semi‐domesticated and effective predator control along with low harvest rates results in high reindeer densities (Tveraa et al. 2003). In spring, the reindeer are herded between inland winter pastures and coastal summer pastures, which are more intensively grazed throughout the summer compared to the pastures along the migratory path (Bråthen et al. 2017). This contrast in grazing intensity and the wide distribution of reindeer in Fennoscandia offers a unique opportunity to study long‐term impacts of herbivores on plant chemical composition at the landscape scale.

In this study, we investigated variation in the chemical composition of tundra plants in response to different temperatures and grazing regimes over the summer season. We quantified plant nitrogen (N) and phosphorus (P) content, which are essential nutrients composing nucleotides, proteins and nucleoside triphosphates and are thus necessary for herbivores' growth, reproduction and survival (Mattson 1980; Parker et al. 2009; Sterner and Elser 2002). We also measured plant defense compounds, particularly silicon (Si), which is present in hard structures that wear herbivore mouth parts and reduce plant digestibility, and phenolics (Ph) which can act as toxins and digestibility reducers, thus decreasing the nutrient uptake of the herbivores (Hartley and DeGabriel 2016; Robbins et al. 1987; Vicari and Bazely 1993).

We sampled plant species across gradients of grazing intensity and summer temperature in northern Norway, with an early and late summer sampling event. It is well known that plant chemical composition varies between species (Murguzur et al. 2019; Smis et al. 2014; Soininen et al. 2013; Thomas et al. 2020), but also that species from the same plant functional group (hereafter PFG) tend to have similar chemical composition (e.g., Murguzur et al. 2019; Hodson et al. 2005). The phenological development of plants throughout the growing season also causes the chemical composition to vary, from small, young, and nutritious shoots in early summer to larger, older and less nutritious shoots later in summer (Iversen et al. 2014; Mattson 1980; Turner et al. 2025).

**FIGURE 1:**
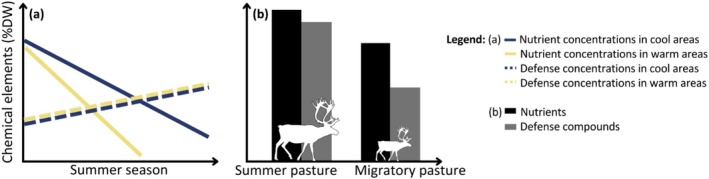
Schematic representation of the hypothesis for the responses of plant nutrient (N, P) and defense compound (Si, Ph) concentrations to long‐term temperature and long‐term grazing effects. (a) Expected variations in nutrients (plain) and defense compound (dotted) concentrations throughout the summer season. (b) Expected pattern of variation in nutrient and defense compound concentrations in heavily grazed (summer pastures) versus lightly grazed (migratory pastures) areas.

Accordingly, we expected pronounced variation in the chemical composition of plants between species, PFGs and over the summer season. Following Doiron et al. (2014), we hypothesized that plants from warmer areas would have a sharper nutrient decline throughout the summer season (Figure 1a). Furthermore, we hypothesized that the long‐established difference in grazing regimes between migratory and summer pastures has shaped the plant chemical composition of the tundra. We expected plants to be more heavily defended in intensely grazed summer pastures (Massey et al. 2007) and to have higher nutrient concentrations due to herbivore fertilization (Barbero‐Palacios et al. 2023; Bardgett and Wardle 2003) when compared to less intensively grazed migratory pastures (Figure 1b). We also explored whether species from different PFGs differed in how their chemical composition responded to seasonality and grazing pressure.

## 2 Materials and Methods

### 2.1 Study System

**FIGURE 2:**
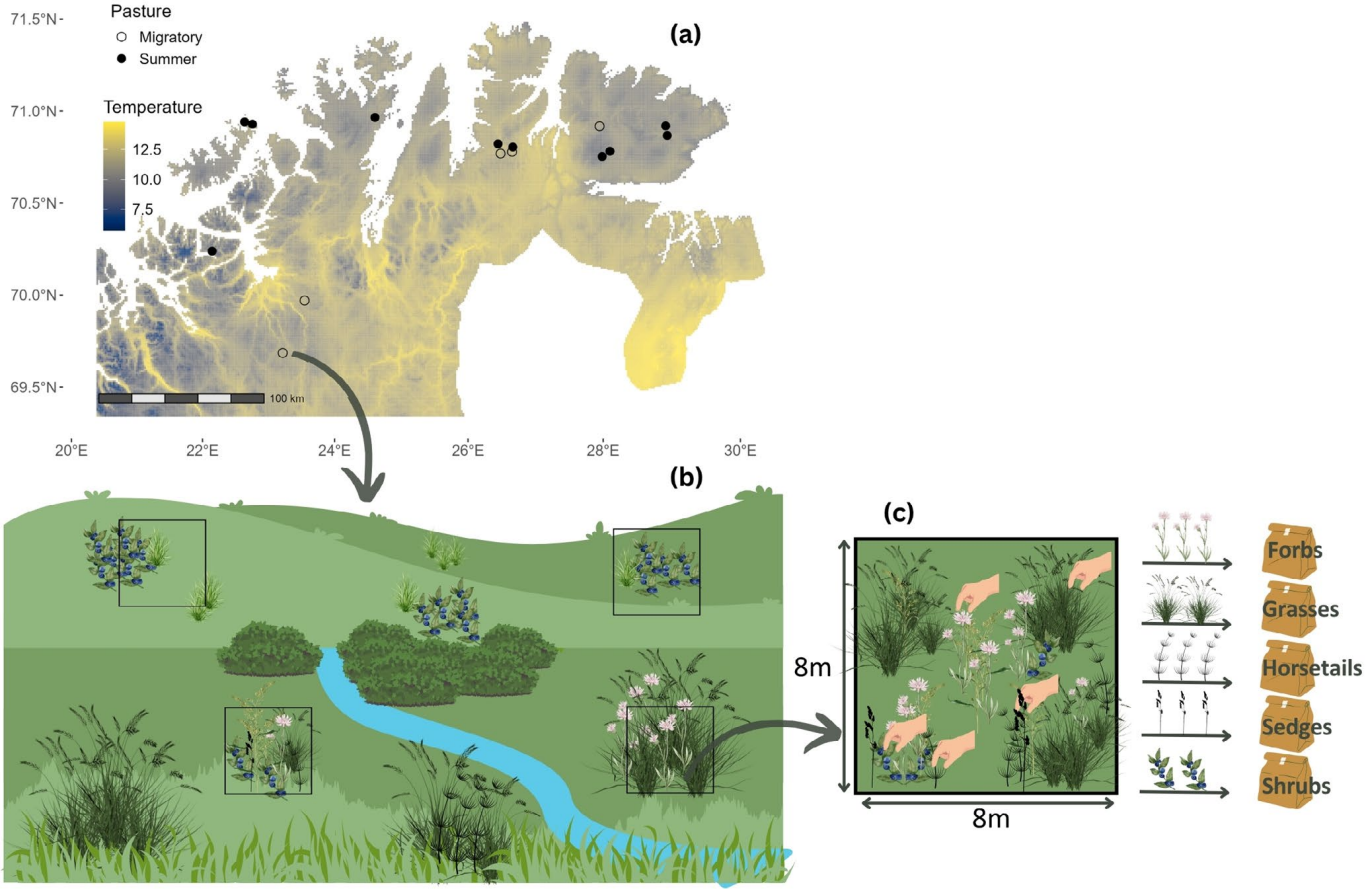
Study design. (a) Heat map displaying the mean July temperatures in the study region along with our 15 sampling sites in summer (filled circles) and migratory (empty circles) pastures. (b) Schematic representation of a sampling site in a summer pasture with 4 plots, 2 in the dwarf shrub heath and 2 in the grassland. (c) Schematic representation of a grassland plot, where the most abundant species of each PFG present is sampled.

Our study region is located in the counties of Troms and Finnmark, in Northern Norway and spans over 140 km from North to South and 285 km from West to East (Figure 2a). The Western part of the region is characterized by steep hills with peaks reaching 1200 m.a.s.l., deep valleys, and narrow fjords, while the eastern part is flatter, with plateaus of 300–500 m.a.s.l. The region has a climatic gradient with colder summer climate along the coast and towards the east, warmer summer climate inland and towards the west, as well as topographically induced variations in temperature (Karlsen et al. 2005). Summary statistics describing reindeer densities and abiotic factors (temperature, precipitation, type of bedrock) in the different parts of our study system are given in the Table S1. Common vegetation types in the study region are dwarf shrub heaths, grasslands, mountain birch forests, and wetlands. In this study, we focus on vegetation types important for reindeer: grasslands which constitute nutritious forage, and dwarf shrub heaths which are common at higher altitudes in reindeer summer pastures (Bråthen et al. 2017; Skarin et al. 2008). Dwarf shrub heaths are dominated by *Empetrum nigrum* , *Betula nana* , *Salix herbacea* , and *Vaccinium* spp. Grasslands are common along river valleys and often have a high biodiversity. They include many PFGs like forbs (*Alchemilla spp*, *Bistorta vivipara, Rumex acetosa, Viola sp*), grasses (*Anthoxanthum nipponicum, Avenella flexuosa, Calamgrostis sp, Deschampsia cespitosa*), sedges (*Carex spp*), and tall shrubs such as *Salix spp* (plant names follow the Pan‐Arctic Flora; http://panarcticflora.org/).

The coast of Northern Norway forms a natural border for the reindeer summer pastures and a combination of natural barriers and fences divides the foraging areas into management districts. The migratory pastures connect inland winter pastures with coastal summer pastures and are used by reindeer during the seasonal migrations twice a year: first in early spring when the ground is still mainly covered in snow, and later in the fall when the plants are senescing. In addition to reindeer, the large herbivore community in this area includes moose ( *Alces alces* ), domestic sheep ( *Ovis aries* ), and, in the larger valleys, small populations of roe deer ( *Cervus elaphus* ). The community of small vertebrate herbivores includes ptarmigans ( *Lagopus lagopus* and *Lagopus muta* ), hares ( *Lepus timidus* ) and small rodents (e.g., *Lemmus lemmus, Microtus oeconomus, Clethrionomys rufocanus*).

### 2.2 Study Design

We selected 10 sampling sites in summer pastures and 5 in migratory pastures (Figure 2), covering eight semi‐domesticated reindeer herding units and spanning a 2.5°C gradient in mean July temperature (climatic normals, Norsk KlimaServiceSenter). Plants were collected during summer 2011 and 2012, both in early July and in late August to capture the seasonal variations in chemical composition. At each sampling site, two plots were sampled in each focal vegetation type (Figure 2b). In two sampling sites no heath was present and thus only grassland plots were sampled (Figure 2b). The minimum distance between two adjacent plots was 100 m, and the plots' dimensions were 8 × 8 m. The field sampling for our study was done in parallel with the Ecofinn project, and therefore has overlapping study design with Bråthen et al. (2017).

### 2.3 Plant Samples

In each plot, the most abundant species from each PFG present—forbs, grasses, sedges, horsetails and shrubs—based on a visual estimate, were selected for harvest (Figure 2c). Plant species were selected solely based on their abundance, regardless of their palatability for reindeer. If several species from the same PFG were deemed equally abundant, they were all sampled and sorted separately. For each species, green, non‐woody parts of several aboveground shoots were sampled to acquire enough plant material for chemical analyses. Minimum sample size for each species was an estimated 2 mg dry weight. In total, 43 different plant species across 56 plots were harvested. Some of the samples have previously been included in research projects with the goal of developing Near‐infrared reflectance spectroscopy (NIRS) as a method to analyze leaf nutrients and defense compounds (Murguzur et al. 2019; Smis et al. 2014).

After collection, plant samples were put in paper bags and frozen. In the laboratory, samples were oven‐dried for a minimum of 72 h at 75°C. Several individuals per species and plot were mixed into one sample to ensure there was about 30 mg of dry material to be pulverized with a ball mill (Mixer Mill, MM301; Retsch GmbH and Co. Haan, Germany). After obtaining a fine and homogeneous plant material, 1056 plant samples were chemically analyzed for N, P, and Si using an elemental analyzer or colorimetric analysis, as presented in Murguzur et al. (2019) and Smis et al. (2014). In addition, 528 samples were analyzed with a NIR spectrometer (FieldSpec 3, Asd Inc., Boulder, Colorado) as described in Murguzur et al. (2019). Further details about the chemical extraction and the NIRS methodology are provided in the Texts S1 and S2. N, P, and Si concentrations (in percentage dry weight) were estimated from the NIR spectral data using calibration models established in Murguzur et al. (2019) and Smis et al. (2014). In addition, P content was estimated using the calibration model available in the Text S3 and Figure S1, and we summarize the average and range of chemical concentrations per species in the Table S3 (Murguzur et al. 2019; Petit Bon, Böhner, et al. 2020; Smis et al. 2014).

Due to the different focus of the different research projects that generated the data, not all samples were analyzed chemically for all chemical compounds (N, P, Si, and Ph), creating discrepancies in the number of samples available for each chemical compound (see Table S2).

### 2.4 Temperature Data on the Study Region

We used the average July temperature from 1991 to 2020 as our measure of summer temperatures at the sampling sites. The data was obtained from a raster map produced by the Norwegian Meteorological Institute with a one‐kilometer pixel size (https://thredds.met.no/temperature/tm_normal_jul_1991‐2020.nc). This is a “climate normal”, that is a three‐decade average that informs about long‐term differences between sites rather than annual fluctuations. Consequently, samples collected in 2011 and 2012 were attributed the same local temperature in our analysis (see Table S1). We extracted the temperatures at the sampling site locations from the raster map with the “sf” package (Pebesma 2018). The site‐specific average July temperatures varied between 9.9°C at the coolest site and 12.4°C at the warmest site.

### 2.5 Statistical Analysis

All the statistical analyses were performed using R statistical software (v4.3.1; R Core Team 2023).

We first explored patterns of variation between species with respect to average nutrient and defense compound concentrations using a principal component analysis (PCA) as implemented in the “*vegan*” package (Oksanen 2022). We calculated the mean N, P, Si, and Ph content for each plant species across all sampling sites and performed a PCA on these means to explore patterns of covariation in nutrient and defense compound concentrations associated with plant functional groups.

We continued by exploring patterns of variation in nutrient and defense compound concentrations with respect to plant functional group, season, vegetation type and grazing intensity using simple bivariate analyses. To evaluate the relative importance of the season, PFG, species within PFG and sampling site, we fitted these predictors as random effects in models describing the plot‐level plant species variation of the chemical compound concentrations (N, P, Ph, Si). In our study design, the sampling site encompasses both variation with respect to grazing intensity and climate. The models were fitted using restricted maximum likelihood using the lmer function in the “*lme4*” package in R (Bates et al. 2015). For ease of interpretation, we present the variance of the random factor as the proportion of the sum of variances for each chemical compound.

Finally, to evaluate our hypotheses with respect to herbivory and the impact of temperature on the seasonal change in the concentrations of N, P, Si, and Ph we fitted linear mixed models (LMM) using restricted maximum likelihood estimation with the lmer function in the “*lme4*” package (Bates et al. 2015). As above, we fitted separate models for each compound (N, P, Si, Ph). As fixed predictor variables we used the variables reindeer grazing pressure (two‐level factor distinguishing migratory and summer pastures), season (two‐level factor that separates early and late summer), temperature at the sampling site (centered around the mean temperature across all sampling sites to facilitate interpretation of intercepts), as well as the interaction between the season and site temperature. We added the variables ‘sampling site’ and ‘species’ as random effects to account for both spatial and species‐specific variations in the different compounds. Expanding on this model, we also evaluated the effect of the vegetation type (two‐level factor that separates dwarf shrub heath and grassland), the effect of variation in reindeer densities on the summer pastures (see Bråthen et al. 2017) and the potential interaction between season and grazing pressure. We found no evidence for any strong patterns with respect to the vegetation type, reindeer density or interaction between season and grazing pressure (*p* > 0.05) and have not included these analyses in the result section (see Table S4, Figures S2 and S3).

## 3 Results

**FIGURE 3:**
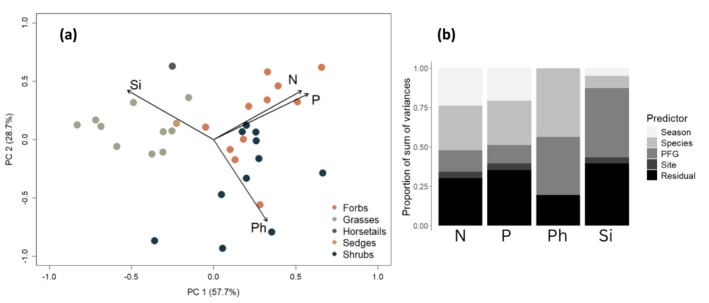
Representations of the variation in the chemical composition of tundra plants. (a) PCA biplot representing the mean chemical composition for each species within different PFG. (b) Variance in chemical compound concentrations explained by the main predictor variables: Season, species, PFG and sampling site, fitted as a random effect model. The sum of variances for N was 0.781, for P was 0.0139, for Ph was 10.6 and for Si was 0.709.

The PCA analysis suggested strong covariation between the nutrient concentrations, that is species with high N content having also high P concentrations (Figure 3a). The first PCA axis (57.7% of the total variance) suggested a weak negative relationship between Si and the nutrients (N, P). In contrast, variation in the concentration of Ph was associated with the second axis (28.7% of the total variance) and appeared independent of N and P concentrations but negatively related to Si. Grass species were associated with high levels of Si and relatively low levels of nutrients. Forbs and shrubs showed substantial variation in both N, P, and Ph. Sedges and horsetails seemed to group close to the grasses in the PCA axes.

The random effect models suggested that the spatial variation associated with the sampling site contributed little to the variation in nutrient and defense compound concentrations (Figure 3b). Variation in nutrient (N and P) concentrations was mainly due to variation between species within PFGs and the time of the season the sampling was done. Variation associated with PFG was intermediate for nutrient concentrations. The defense compounds differed from the nutrients in that a large part of the variance in concentration was due to variation between the PFGs, and that sampling season explained little variation. Ph concentrations showed substantial variation also between species within PFGs, while the variation between species within PFGs was relatively small with respect to Si concentrations.

**FIGURE 4:**
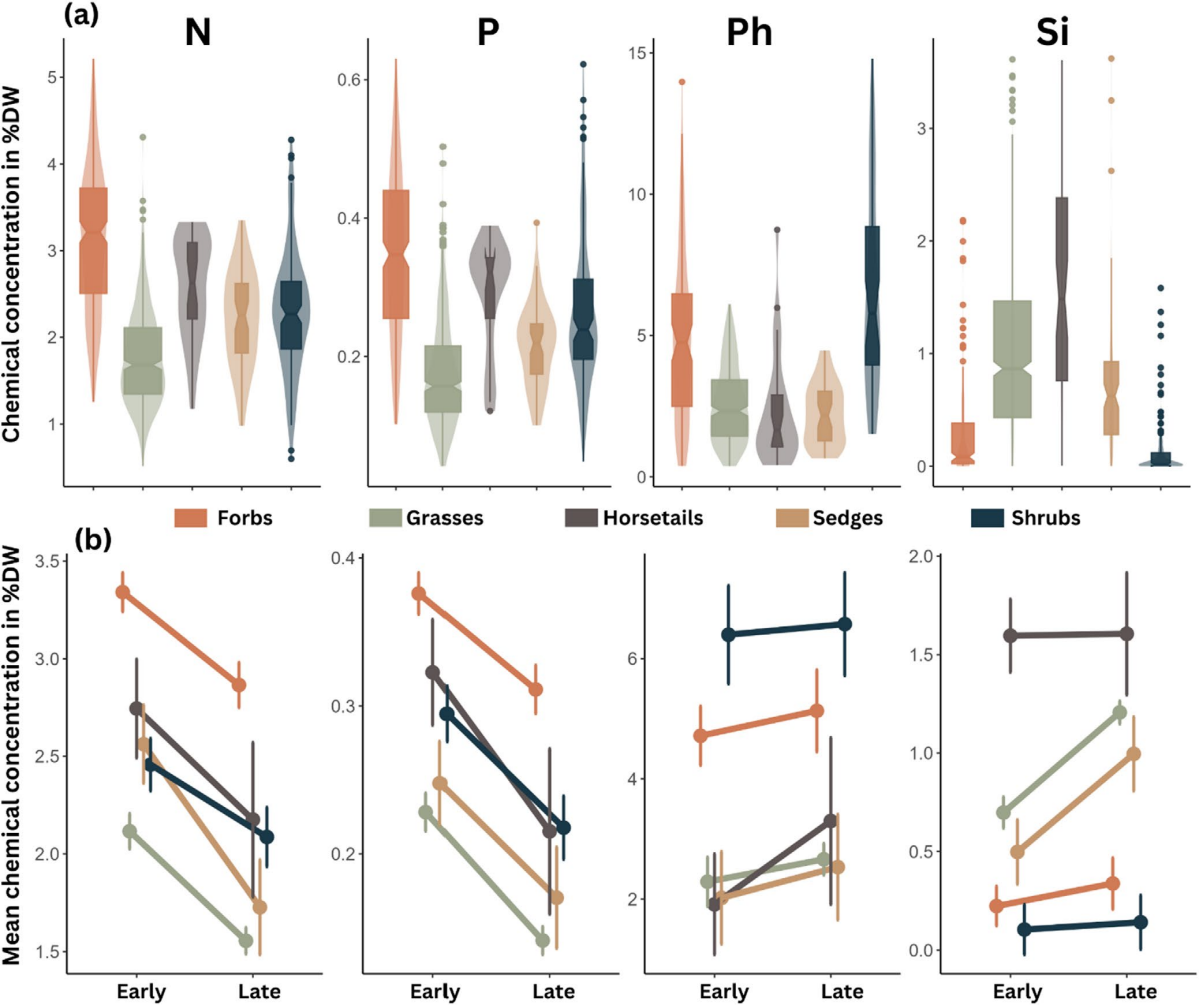
Characteristic chemical composition and seasonal variation per PFG in percent dry weight (%DW). (a) Distribution of chemical compounds represented as a violin plot overlaid with boxplots for each PFG. The notches represent the median, the whiskers 1.5 times the interquartile range, and outliers are shown as points. The thickness of the box plots is proportional to the number of samples for each PFG. (b) Mean change in chemical content from early to late summer for each PFG. Means were estimated with a linear model fitting the chemical response to the interaction of PFG and season. Error bars represent the 95% confidence interval around the mean.

The distribution of the chemical compounds within each PFG as shown in Figure 4a further supported the patterns of the PCA (Figure 3a). Forbs were generally more nutrient‐rich while grasses were less nutrient‐rich, and horsetails sedges and shrubs had intermediate concentrations of nutrients (N and P). Most forbs and shrubs had very low concentrations of Si, while grasses, horsetails and sedges tended to have higher concentrations. In contrast, phenolics were typically found at higher concentrations in forbs and shrubs than in grasses, horsetails and sedges (Figure 4a). As expected, average nutrient concentrations were lower late in the summer season than early across PFGs (Figure 4b), while the seasonal change in defense compounds was not consistent across PFGs. For many PFGs the data suggested a very weak positive trend or no seasonal trend in the defense compound concentrations, supporting the finding in the random effect model (Figure 3b). In contrast, there was a pronounced seasonal increase in Si concentrations in grasses and sedges.

**FIGURE 5:**
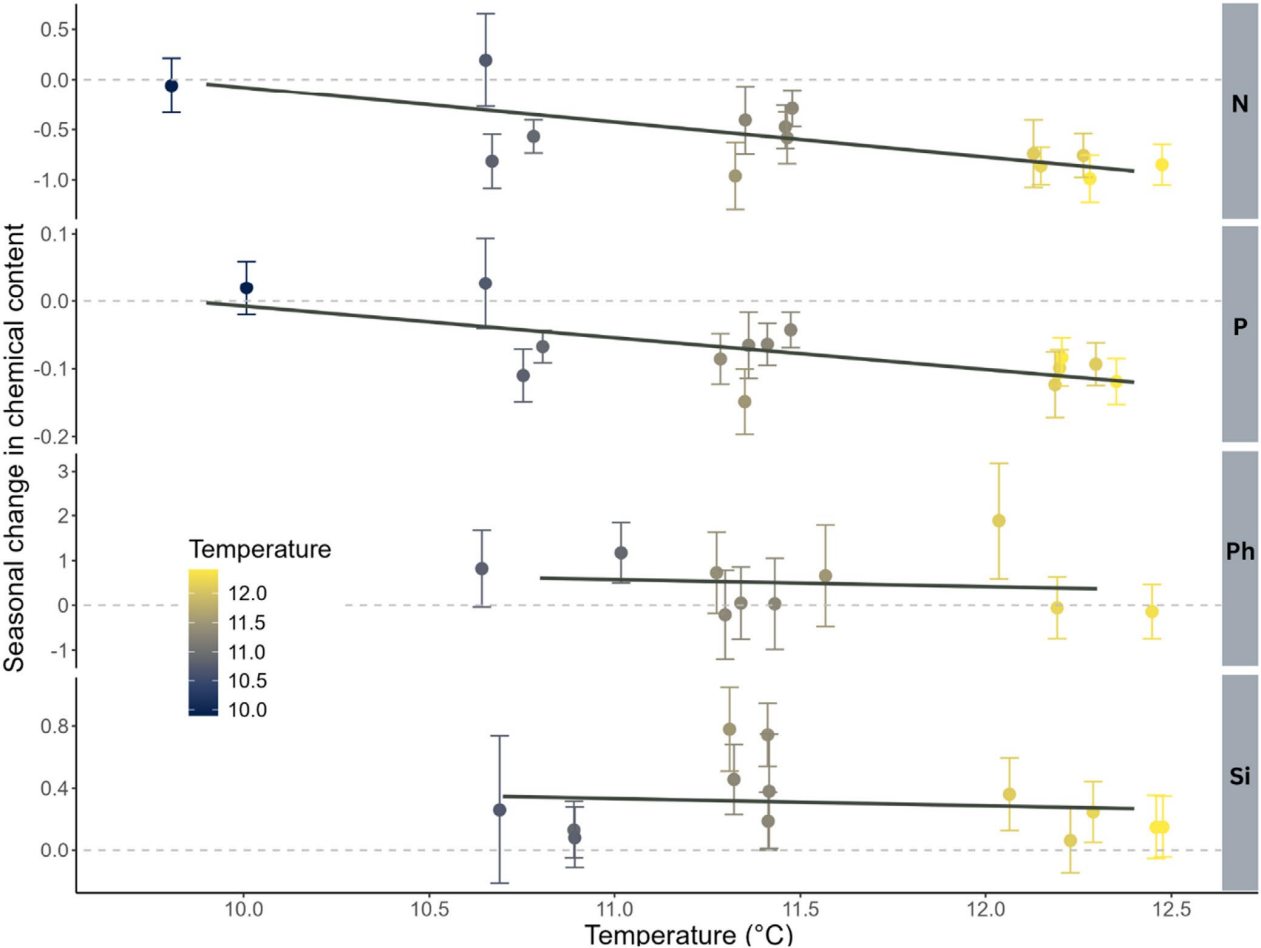
Seasonal change in chemical concentration. Average seasonal change in chemical concentration for N, P, Si, and Ph in relation to the average summer temperature at each site. The points give the average seasonal change in chemical concentration across species at each study site (±1 SE) as estimated using a linear mixed effects model with sampling site, season and the interaction between sampling site and season fitted as fixed effects, and species fitted as a random effect. The lines are used as a visual aid to show the temperature trend.

The linear mixed models suggested seasonal changes across species and sites in both nutrients and defense compounds (Table 1). The concentrations of N and P decreased while Si and Ph increased over the summer. These analyses also suggested a positive relationship between N concentrations and temperature early in the summer season, and that warmer sites had a steeper seasonal decline in N concentrations than colder sites (Table 1, Figure 5). In contrast, there was no strong evidence for a positive relationship between P concentrations and temperature early in the summer season, but as for N, warmer sites had a steeper decline in P concentrations over the summer (Table 1, Figure 5). The finding of a steeper seasonal decline supports our initial hypothesis. However, the processes generating these patterns were different for N and P. The steeper seasonal decline in average N concentrations at warmer sites was mainly due to higher early summer N concentrations at warm sites, with average N concentrations being similar in warm and cold sites in late summer (estimated temperature slope for late summer average N concentrations = −0.05, 95% CI = [−0.21, 0.11], see Table S5, Figure S4). In contrast, average P concentrations were similar, independent of temperature, in early summer but showed a negative relationship to temperature in late summer (estimated temperature slope for late summer average P concentrations = −0.02, 95% CI = [−0.045, −0.001]). We found no strong evidence for summer temperature to affect defense compound concentrations, or any evidence for nutrient and defense compound concentrations to vary in association with reindeer grazing intensity (Table 1).

**TABLE 1:** Parameter estimates and associated 95% CI from generalized linear mixed models for N, P, Si, and Ph concentrations with grazing intensity, temperature, season and the interaction between temperature and season fitted as fixed effects.

Predictors	N		P		Si		Ph	
	Estimates	CI	Estimates	CI	Estimates	CI	Estimates	CI
Intercept	2.55	2.29–2.81	0.3	0.26–0.33	0.42	0.20–0.64	4.15	3.13–5.17
Grazing intensity (Summer pasture)	0.03	−0.20 to 0.26	−0.02	−0.05 to 0.01	−0.07	−0.28 to 0.15	−0.12	−0.44 to 0.19
C Temp	**0.25**	**0.09–0.40**	0.01	−0.01 to 0.03	−0.13	−0.32 to 0.06	−0.1	−0.47 to 0.26
Season (Late season)	**−0.64**	**−0.70 to −0.57**	**−0.08**	**−0.09 to −0.07**	**0.27**	**0.21–0.34**	**0.3**	**0.01–0.59**
C Temp × Season (Late season)	**−0.3**	**−0.39 to −0.20**	**−0.04**	**−0.05 to −0.02**	−0.03	−0.14 to 0.08	−0.3	−0.76 to 0.16
Random effects								
*σ* ^2^ Species	0.330		0.006		0.209		8.205	
*σ* ^2^ Sampling sites	0.030		0.001		0.026		0	
*σ* ^2^ Residuals	0.224		0.005		0.279		2.042	
Observations	948		949		1189		528	

*Note:* Species and sampling site were fitted as random effects (*σ* ^2^). Observations give the number of samples included in each model. Statistically significant effects are shown in bold.

## 4 Discussion

To our knowledge, this is the first study that uses a large spatial sampling design to assess long‐term impacts of herbivory and climate on plant nutritional quality (i.e., both defense and nutrient concentrations). In our Arctic tundra system, we find substantial variation in nutrient and defense compound concentrations between species, and part of this between‐species variation was associated with plant functional groups, confirming findings in previous studies (Murguzur et al. 2019; Smis et al. 2014; Thomas et al. 2019). We also document strong seasonal decreases in the concentrations of N and P and weaker seasonal increases in concentrations of Si and Ph over the summer (Figures 3b and 4b, Table 1). Consistent with our expectation, we found a steeper seasonal decline in nutrient concentrations in warmer sites than in cooler sites, while defense compounds seemed not to be affected by the temperature. Contrary to our expectations (Figure 1b), we found no evidence for long‐term effects of reindeer grazing intensity on the average chemical composition of the plants. Our results indicate that the quality of reindeer summer forage is strongly shaped by species identity, functional composition and seasonal variation, which in turn is modified by temperature.

### 4.1 Effect of Temperature and Its Potential Influence on the Seasonal Changes in Plant Chemical Content

The variation in temperatures observed in this study between sampling sites (2.5°C) was similar to the temperature differences between control and warm treatments in experimental studies (Figure 5, Doiron et al. 2014; Elmendorf et al. 2012; Tolvanen and Henry 2001) and was lower than the increase in Arctic mean temperatures expected by 2050, with estimates ranging from 3.0°C to 4.6°C under different climatic scenarios (Wang 2021). Consistent with previous short‐term experimental studies (Doiron et al. 2014), we found a steeper seasonal decline in N and P concentrations with warmer temperatures (Table 1, Figure 5). For N, this pattern was mainly due to a positive relationship between temperature and average N concentrations early in the summer season. This pattern is inconsistent with what is seen in experimental warming studies (Doiron et al. 2014) where N concentrations in the early season tend to be similar across temperature treatments, and differences develop through the summer. The pattern found for P concentrations is more aligned with the findings in Doiron et al. (2014), as the temperature effect on the seasonal change in P concentrations was mainly due to differences that develop over the summer season. Comparisons with other studies that do not take the strong seasonal decline in nutrient concentrations into account (Bjorkman et al. 2018; Reich and Oleksyn 2004) or only sample the plants once in the middle of the summer (Petit Bon et al. 2023) are more difficult as the conclusions of these studies may depend on when the sampling took place in the season. Clearly, the strong seasonal variation in nutrient concentrations makes the design of nutrient concentration studies difficult, and we may only speculate that the patterns detected for N in our study may be due to temperature differences in the phenological development of plants sampled at a similar time of year. Access to additional data on the onset of spring for each location would have mitigated the limitation that the observed pattern for N may result from variations in the plants' phenological states. We found no evidence for Si, or Ph concentrations being affected by temperature. Our results suggest that defense compounds in general are robust to temperature changes (see also Nybakken et al. 2011) although there might be species‐specific variations in Ph concentrations in response to warming, as found in Nybakken et al. (2008) and Zhou et al. (2022).

### 4.2 Long‐Term Effect of Grazing on Plant Chemical Composition

We found no evidence for an effect of reindeer grazing intensity on plant chemical composition. We expected plants from summer pastures to have higher concentrations of nutrients and defense compounds than plants from migratory pastures due to long‐term differences in fertilization and grazing pressure (Figure 1b). In Alpine meadows, long‐term grazing by domestic cattle was reported to increase the concentrations of N and P in plants (Niu et al. 2016); defense compounds were not studied. Short‐term experiments in tundra habitats have also reported a general increase in the nutrient concentration in plants in response to small rodent activity (Petit Bon, Gunnarsdotter Inga, et al. 2020; Tuomi et al. 2019), reindeer grazing (Petit Bon, Gunnarsdotter Inga, et al. 2020) and experimental goose grubbing (Petit Bon et al. 2023) and a general increase in the quality of grasses in the pastures grazed by small rodents or reindeer (Petit Bon et al. 2022). Although numerous studies have investigated the influence of climate and herbivory on plant nutrient concentrations, there is a significant gap in our understanding of how herbivory affects the concentrations of defense compounds in tundra plants. Indeed, studies on plant defenses generally focus on either short‐term within‐species induction or a long‐term change in plant community composition, making our approach of long‐term herbivory impacts on defense levels unique also beyond tundra vegetation (but see Didiano et al. 2014). Distinguishing between different mechanisms of herbivore impacts on defenses represents a crucial area for further investigation, and in our case, would provide a more complete picture of the quality of the forage available to reindeer in their summer pastures. While the effect of reindeer grazing on plant chemical composition was small, we acknowledge that reindeer grazing may affect the chemical composition of the pastures through its influence on the plant community composition, whether by maintaining the vegetation in its current state (Bråthen et al. 2017; Post and Pedersen 2008; Ramirez et al. 2024), or by modifying the biomass of the species present (Bernes et al. 2015; Bråthen et al. 2007; Ravolainen et al. 2011; Sundqvist et al. 2019; Zhang et al. 2023).

### 4.3 Variation Between Plant Functional Groups

The PFGs monitored in this study showed characteristic chemical differences (Figures 3a and 4a) and had relatively similar responses to seasonal changes. Across all PFGs, the concentrations of N and P decreased, which is explained by the relocation of nutrients from the leaves to the stems and roots towards the end of the summer season (Arndal et al. 2009; Kelsey et al. 2023; Tolvanen and Henry 2001; Westergaard‐Nielsen et al. 2021). The increase in the concentrations of defense compounds was less clear and PFG‐dependent, likely explained by the negative correlation observed between Si and Ph (Figure 3a) which highlights a tradeoff in plant defense strategies (de Tombeur et al. 2021). Consequently, PFGs like grasses and sedges which are typically rich in Si (Hodson et al. 2005; Smis et al. 2014; Soininen et al. 2013) had a large increase in Si and less so in Ph throughout the season (Figure 4b). Grasses have a great capacity to accumulate Si (Hartley and DeGabriel 2016; Vicari and Bazely 1993) and considering that the long‐term reindeer grazing had no strong effect on plant defense concentrations, this seasonal increase may be more likely attributed to an accumulation of Si rather than an induced defense response of the plant (Mcnaughton and Tarrants 1983). The seasonal component explained very little of the variation in Ph concentrations (Figure 3b). In a study about mountain birch, Riipi et al. (2002) reported that Ph increased across the summer season, with a peak in early June and at senescence, in late September. Our period between seasonal sampling being shorter, we might not have captured the whole seasonal variation in Ph, which could explain the seemingly mild increase in Ph concentrations towards senescence. The trait composition of plants in the tundra system is highly context dependent (Kemppinen and Niittynen 2022) and other abiotic (moisture, wind exposure) or biotic (small rodent disturbance, competition, intra‐specific variation) factors could also have influenced the concentrations of both nutrients and defense compounds across PFGs.

## 5 Conclusion

With the use of NIRS methodology we obtained data on the concentrations of nutrient and defense compounds in plants sampled at an unprecedented spatial scale, thus providing novel insights into the variation in plant nutritional quality in the tundra ecosystem. We find that large‐scale variation in average summer temperatures and reindeer grazing pressure has relatively small effects on the spatial variation in plant chemical composition. The quality of the vegetation in an area is therefore likely to be mainly determined by the plant species present, including their abundances and species‐specific chemical signatures. In addition, we note that the seasonal decrease in plant quality in Arctic tundra is a main factor determining the seasonal change in the quality of the forage available to Arctic herbivores. Warmer temperatures are likely to enhance plant biomass production (Elmendorf et al. 2012; Van Der Wal and Stien 2014; Walker et al. 2006) and combined with a seasonal decrease in plant nutrient concentrations, these processes may lead to the dilution of essential nutrients in the vegetation, resulting in a decline in the quality of the resources available for Arctic herbivores like reindeer (Petit Bon et al. 2023). As species and plant functional diversity uphold the variation in forage quality, monitoring changes in species composition of tundra plant communities and their quality over time is fundamental to understanding and managing these systems. Future research should combine analysis of both plant chemical composition and plant abundance to estimate the foodscape available to the herbivores throughout the summer season and its responses to climate change.

## Author Contributions

**Fanny Berthelot:** data curation (lead), formal analysis (lead), investigation (lead), visualization (lead), writing – original draft (lead), writing – review and editing (lead). **Audun Stien:** formal analysis (supporting), investigation (supporting), methodology (supporting), supervision (lead), validation (equal), writing – review and editing (equal). **Eeva M. Soininen:** formal analysis (supporting), investigation (equal), supervision (equal), validation (equal), writing – review and editing (equal). **Torkild Tveraa:** supervision (equal), validation (equal), visualization (equal), writing – review and editing (equal). **Hanna Böhner:** data curation (equal), methodology (equal), software (equal), validation (equal). **Kari Anne Bråthen:** conceptualization (lead), formal analysis (equal), funding acquisition (equal), investigation (equal), methodology (lead), project administration (lead), resources (lead), supervision (equal), validation (equal), writing – review and editing (equal).

## Conflicts of Interest

The authors declare no conflicts of interest.

## Supporting information


**File S1:** ece372500‐sup‐0001‐Supinfo.docx.

**Data S1:** ece372500‐sup‐0002‐DataS1.txt.

**Data S2:** ece372500‐sup‐0003‐DataS2.R.

## Data Availability

The data and the R script used for the analysis are stored and available on DataverseNO (https://dataverse.no/).
