# Supplementary material for: Are Large‐Scale Differences in Temperature and Reindeer Management Regime Affecting the Quality of Reindeer's Summer Forage?
Source: Ecol Evol. 2025 Nov 15;15(11):e72500. doi: 10.1002/ece3.72500 (PMC12619109; doi:10.1002/ece3.72500)
Supplement: Supplementary file 1 — File S1: ece372500‐sup‐0001‐Supinfo.docx. [file ECE3-15-e72500-s003.docx]

**Supporting Information for:**

**Are large-scale differences in temperature and reindeer management regime affecting the quality of reindeer’s summer forage?**

**Fanny Berthelot, Audun Stien, Eeva M. Soininen, Torkild Tveraa, Hanna Bohner, Kari Anne Bråthen**

**Table of Contents:**

| **Table 1: Summary of reindeer density and abiotic factors per sampling site** | Page 2 |
| --- | --- |
| **Text 1: Methodological information about chemical analysis** | Page 3 |
| **Text 2: Methodological information about NIRS analysis** | Page 4 |
| **Table 2: Sample summary of chemical analysis** | Page 5 |
| **Text 3: Calibration of phenolics using NIRS** | Page 5 |
| **Figure 1: Relationship between observed and predicted total phenolic**  **concentrations** | Page 6 |
| **Table 3: Summary of the sample size, mean and range for each chemical element for all species covered in the study.** | Page 7-8 |
| **Table 4: General model outputs testing for the additional effects of habitat, reindeer density and rejuvenation effect** | Page 9 |
| **Figure 2: Chemical composition of different functional groups in grasslands and**  **heaths.** | Page 10 |
| **Figure 3: Average chemical content (N, P, Si and Ph) per species.** | Page 11 |
| **Figure 4: Average chemical content (N, P, Ph and Si) in early and late season along**  **a temperature gradient.** | Page 12 |
| **Table 5: Parameter estimates and associated 95% CI from generalized linear mixed models for N and P concentrations fitted as response variable, after relevelling.** | Page 13 |

**Table 1: Summary of the ecological context, reindeer density and abiotic factors of the sampling sites.**

¤ = Total reindeer population size from <https://www.reinbase.no/>, divided by the district area in km^2^ from Ressursregnskap for reindriftsnæringn (2013).

* = Data from <https://www.met.no/>.

** = Data from <https://www.norgeskart.no/>.

$ = Data from <https://www.ngu.no/>

**Text 1: Additional methodological information about chemical analysis for N, P and Si following Murguzur et al. (2019) & Smis et al. (2014).**

Respectively 117 and 29 samples were analyzed for N and P content by a CNS elemental analyser (Flash 2000 Organic elemental analyser, Thermo Scientific, UK), and 312 and 323 samples were analyzed for N and P content by colorimetry using a segmented flow analyser. For all chemical analysis the recovery was at least 90% of Certified Reference Material (BCR-129 Institute for Reference Materials and Measurements at the European Commission Joint Research Centre). These data were first published by Murguzur et al. 2019.

At least 577 samples were analysed for Si content, and were obtained by a colorimetric analysis, preceded by a chemical digestion. Plant biogenic silica (BSi) was extracted with the wet alkaline (0.1M Na_2_CO_3_) method which is suitable for BSi analysis of plant material (Meunier et al. 2013). About 30 mg of pulverized material was incubated for 4 h in 0.1M Na_2_CO_3_ at 80°C (DeMaster, 1991) and 10 ml of the extract was filtered (Chromafil® A-45/25, pore size of 0.45μm). After extraction, samples were stored in the dark at 3°C and went through a colorimetric analysis for extracted dissolved Si (DSi) within maximum two weeks, using a SKALAR SA 1500 colorimeter. Blank extractions were subtracted to account for DSi release from recipients and chemicals. These data were first published by Smis et al. 2014.

References:

DeMaster, D. J. (1991). Measuring biogenic silica in marine sediments and suspended matter. Geophys. Monogr. 63, 363–367.

Meunier, J. D., Keller, C., Guntzer, F., Riotte, J., Braun, J. J., and Anuparna, K. (2013). Assessment ofthe 1%Na2CO3 technique to quantify the phytolith pool.Geoderma 216, 30–35. doi: 10.1016/j.geoderma.2013.10.014

Murguzur, F. J. A., Bison, M., Smis, A., Böhner, H., Struyf, E., Meire, P., & Bråthen, K. A. (2019). Towards a global arctic-alpine model for Near-infrared reflectance spectroscopy (NIRS) predictions of foliar nitrogen, phosphorus and carbon content. *Scientific Reports*, *9*(1). https://doi.org/10.1038/s41598-019-44558-9

Smis, A., Ancin Murguzur, F. J., Struyf, E., Soininen, E. M., Herranz Jusdado, J. G., Meire, P., & Bråthen, K. A. (2014). Determination of plant silicon content with near infrared reflectance spectroscopy. *Frontiers in Plant Science*, *5*(SEP). https://doi.org/10.3389/fpls.2014.00496

**Text 2: Additional methodological information about NIRS analysis**

Pulverized samples of dry plant matter were transformed into tablets (Ø16mm, >1mm thick) by applying 6 tons of pressure with a hydraulic press. The obtained homogeneous and ﬂat vegetal material has a surface that reduces random light scatter, thus reducing random variation in spectral signatures. Since water shows strong absorption patterns in the near-infrared region (Givens, De Boever, & Deaville, 1997), the tablets were oven dried for 2 hours at 50**°**C to remove any potential humidity. They were then stored in a desiccator at room temperature (approx. 20°C) until they were scanned with a hand-held NIRS spectrometer (FieldSpec 3, ASD Inc., Boulder, Colorado, USA) with monochromatic radiation in a wavelength range of 350-2500 nm. The spectra were interpolated to 1 nm intervals based on recordings at 1.4 nm from 350 to 1050 nm and at 2 nm from 1000 to 2500 nm. Wavelength regions with overlap from the sensors (i.e. 350–380 nm, 760–840 nm, 1700–1800nm and 2450–2500 nm) as well as the visible part of the spectrum (380 – 720 nm) were removed (Murguzur et al., 2019). The final spectrums consisted of the average of 3 replicate scans per sample, recorded as absorbance (log 1/R, with R = reflectance). The percentages dry weight of N, P, and Si were calculated from the spectrums using calibrations established in (Murguzur et al., 2019; Smis et al., 2014).

References:

Givens DI, De Boever JL, Deaville ER. The principles, practices and some future applications of near infrared spectroscopy for predicting the nutritive value of foods for animals and humans. Nutr Res Rev. 1997 Jan;10(1):83-114. doi: 10.1079/NRR19970006. PMID: 19094259.

Murguzur, F. J. A., Bison, M., Smis, A., Böhner, H., Struyf, E., Meire, P., & Bråthen, K. A. (2019). Towards a global arctic-alpine model for Near-infrared reflectance spectroscopy (NIRS) predictions of foliar nitrogen, phosphorus and carbon content. *Scientific Reports*, *9*(1). https://doi.org/10.1038/s41598-019-44558-9

Smis, A., Ancin Murguzur, F. J., Struyf, E., Soininen, E. M., Herranz Jusdado, J. G., Meire, P., & Bråthen, K. A. (2014). Determination of plant silicon content with near infrared reflectance spectroscopy. *Frontiers in Plant Science*, *5*(SEP). https://doi.org/10.3389/fpls.2014.00496

**Table 2: Sample summary of chemical analysis.**

Our samples were originally collected for calibrations with NIRS in 2 different studies. Consequently, some of our samples have only been analyzed for only one chemical component (e.g. only Si) out of 4. This table gives an overview of the number of samples which went through different analysis.

|  | **Total No Samples** | **N Samples** | **P Samples** | **Si Samples** | **Ph Samples** |
| --- | --- | --- | --- | --- | --- |
| **Chemical analysis** | 1056 | 422 | 421 | 661 | - |
| CNS | 117 | 117 | 29 | - |  |
| Colorimetry | 900 | 312 | 323 | 577 |  |
| NA | 39 | - | 69 | 84 | - |
| **NIRS analysis** | 528 | 528 | 528 | 528 | 528 |

**Text 3: Calibration of phenols using NIRS**

A subset of the plant samples (n=165) used to develop NIRS calibration models for carbon, nitrogen and phosphorus (Murguzur et al. 2019) were used to also develop a NIRS calibration model for total phenolics.

The plant samples were prepared and analyzed with a NIR spectrometer (FieldSpec 3, Asd Inc., Boulder, Colorado) as described in Murguzur et al. (2019). A subsample of these samples was then used to extract and measure total phenolics using the Folin-Ciocalteau method (Salminen and Karonen 2011, Waterhouse 2002).

Partial least squares regression (PLSR) was used to develop a calibration model for predicting total phenolics from NIR spectral data. The total dataset was split in a calibration set (80% of the samples) and a validation dataset (20% of the samples) using the Kennard-Stone algorithm (Kennard and Stone 1969). The calibration data set was used for developing the model including internal cross-validation and the validation data set was used for testing the final model. A Savitzky-Golay filter (Savitzky and Goaly 1946) was applied to the spectral data before PLSR modelling. The final model was chosen based on an evaluation of the coefficient of determination (R^2^), the number of components (k) and the root mean square of the error of the cross-validation (RMSECV). This model was evaluated on the validation data set by calculating coefficient of determination (R^2^) and the root mean standard error of prediction (RMSEP) (Figure 1).

Figure 1: Relationship between observed and predicted total phenolic concentrations from cross-validation during model development using the calibration data set and from external validation of the final model using the validation data set.

**Figure 1: Relationship between observed and predicted total phenolic concentrations.** Figure issued from cross-validation during model development using the calibration data set and from external validation of the final model using the validation data set.


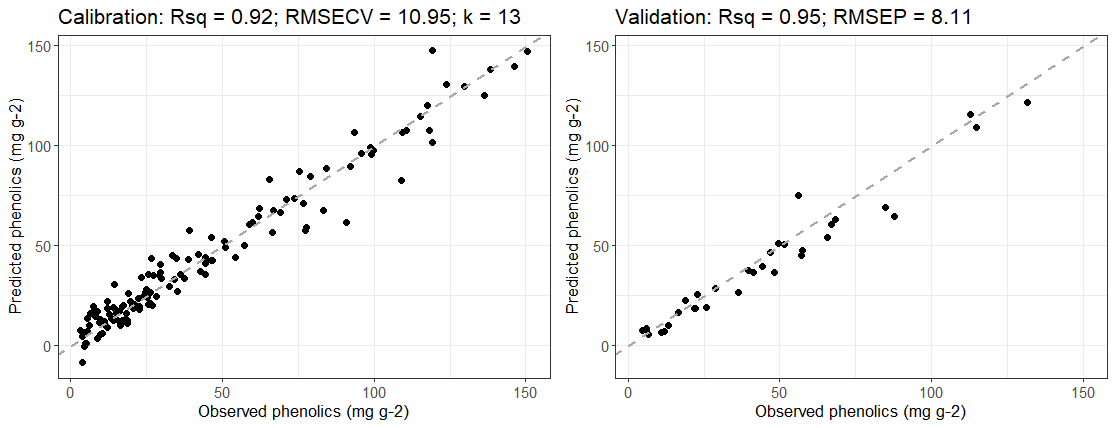


References:

Kennard, R. W., & Stone, L. A. (1969). Computer aided design of experiments. *Technometrics*, *11*(1), 137-148.

Murguzur, F. J. A., Bison, M., Smis, A., Böhner, H., Struyf, E., Meire, P., & Bråthen, K. A. (2019). Towards a global arctic-alpine model for Near-infrared reflectance spectroscopy (NIRS) predictions of foliar nitrogen, phosphorus and carbon content. *Scientific Reports*, *9*(1). <https://doi.org/10.1038/s41598-019-44558-9>

Salminen, J. P., & Karonen, M. (2011). Chemical ecology of tannins and other phenolics: we need a change in approach. *Functional ecology*, *25*(2), 325-338.

Savitzky, A., & Golay, M. J. (1964). Smoothing and differentiation of data by simplified least squares procedures. *Analytical chemistry*, *36*(8), 1627-1639

Waterhouse, A. L. (2002). Determination of total phenolics. *Current protocols in food analytical chemistry*, *6*(1), I1-1.

**Table 3: Summary of the sample size, mean and range for each chemical element for all species covered in the study.**

| **Functional group** | **Nitrogen (% dry weight)** | | | **Phosphorus (% dry weight)** | | | **Silicon (% dry weight)** | | | **Phenolics (% dry weight)** | | |
| --- | --- | --- | --- | --- | --- | --- | --- | --- | --- | --- | --- | --- |
| Species | No samples | Mean | Range | No samples | Mean | Range | No samples | Mean | Range | No samples | Mean | Range |
| **Forbs** |  |  |  |  |  |  |  |  |  |  |  |  |
| *Alchemilla sp* | 53 | 2.553 | 1.43-3.60 | 53 | 0.254 | 0.11-0.52 | 75 | 0.375 | 0.003-1.19 | 42 | 6.222 | 2.27-12.13 |
| *Bistorta vivipara* | 53 | 3.230 | 2.15-4.56 | 53 | 0.386 | 0.14-0.59 | 18 | 0.246 | 0.004-0.2.17 | 8 | 4.552 | 2.54-6.29 |
| *Comarum palustre* | 1 | 2.781 | - | 1 | 0.253 | - | 3 | 0.135 | 0.002-0.34 | 1 | 3.979 | - |
| *Geranium sp* | 13 | 2.483 | 1.25-3.90 | 13 | 0.260 | 0.10-0.49 | 19 | 0.200 | 0.003-1.85 | 7 | 9.877 | 6.61-13.97 |
| *Geum sp* | 0 | - | - | 0 | - | - | 4 | 0.865 | 0.02-1.82 | 0 | - | - |
| *Pyrola sp* | 0 | - | - | 0 | - | - | 1 | 0.027 | - | 0 | - | - |
| *Ranunculus sp* | 4 | 2.234 | 1.96-2.38 | 4 | 0.250 | 0.16-0.33 | 8 | 0.257 | 0.005-1.43 | 2 | 1.667 | 1.49-1.85 |
| *Rumex sp* | 81 | 3.760 | 1.86-5.22 | 81 | 0.408 | 0.23-0.63 | 18 | 0.055 | 0.004-0.30 | 3 | 2.439 | 1.24-3.45 |
| *Saussurea alpina* | 1 | 3.834 | - | 1 | 0.339 | - | 2 | 1.188 | 0.38-1.20 | 1 | 5.718 | - |
| *Solidago virgaurea* | 18 | 2.957 | 1.80-4.67 | 18 | 0.343 | 0.17-0.60 | 39 | 0.218 | 0.01-1.16 | 15 | 2.724 | 0.38-6.38 |
| *Trientalis europaea* | 3 | 2.218 | 1.78-2.82 | 3 | 0.282 | 0.26-0.31 | 18 | 0.196 | 0.06-0.36 | 3 | 4.227 | 3.32-5.17 |
| *Trollius europaeus* | 9 | 2.674 | 1.50-4.21 | 9 | 3.319 | 0.14-0.50 | 18 | 0.129 | 0.002-0.29 | 6 | 1.758 | 0.56-3.38 |
| *Vicia cracca* | 0 | - | - | 0 | - | - | 2 | 0.403 | 0.16-0.65 | 0 | - | - |
| *Viola sp* | 10 | 2.911 | 2.21-3.32 | 10 | 0.389 | 0.14-0.54 | 17 | 0.152 | 0.005-0.63 | 8 | 1.897 | 1.15-2.87 |
| **Grasses** |  |  |  |  |  |  |  |  |  |  |  |  |
| *Agrostis sp* | 14 | 2.136 | 1.13-3.16 | 14 | 0.196 | 0.05-0.31 | 22 | 1.447 | 0.003-3.47 | 10 | 2.158 | 0.80-3.53 |
| *Alopecurus pratensis* | 3 | 1.889 | 1.44-2.69 | 3 | 0.198 | 0.07-0.37 | 5 | 0.483 | 0.08-0.99 | 2 | 0.947 | 0.75-1.15 |
| *Anthoxanthum sp* | 79 | 2.014 | 0.88-3.21 | 79 | 0.215 | 0.06-0.42 | 57 | 0.625 | 0.003-3.33 | 27 | 2.046 | 0.90-2.82 |
| *Avenella flexuosa* | 120 | 1.718 | 0.75-3.05 | 120 | 0.198 | 0.07-0.36 | 140 | 0.534 | 0.01-3.16 | 65 | 2.627 | 0.66-5.53 |
| *Calamagrostis sp* | 50 | 1.702 | 0.92-3.57 | 50 | 0.142 | 0.07-0.32 | 82 | 1.485 | 0.006-3.61 | 39 | 2.366 | 0.88-4.73 |
| *Deschampsia sp* | 96 | 1.767 | 0.59-4.31 | 96 | 0.138 | 0.04-0.39 | 135 | 1.298 | 0.01-3.26 | 85 | 3.722 | 0.41-6.11 |
| *Elymus repens* | 9 | 1.549 | 0.88-1.94 | 9 | 0.154 | 0.08-0.23 | 15 | 1.443 | 0.43-3.06 | 9 | 1.399 | 0.74-2.44 |
| *Elytrigia repens* | 0 | - | - | 0 | - | - | 15 | 1.186 | 0.06-2.94 | 0 | - | - |
| *Festuca ovina* | 2 | 2.143 | 1.73-2.56 | 2 | 0.288 | 0.24-0.34 | 4 | 0.738 | 0.21-0.97 | 2 | 1.007 | 0.72-1.30 |
| *Nardus stricta* | 46 | 1.460 | 0.51-2.22 | 46 | 0.118 | 0.04-0.20 | 89 | 1.518 | 0.02-3.34 | 38 | 1.180 | 0.38-2.85 |
| *Phleum sp* | 54 | 1.633 | 0.98-3.47 | 54 | 0.178 | 0.06-0.50 | 79 | 0.538 | 0.009-1.70 | 35 | 2.243 | 0.45-4.17 |
| *Vahlodea atropurea* | 0 | - | - | 0 | - | - | 1 | 0.529 | - | 0 | - | - |
| **Horsetail** |  |  |  |  |  |  |  |  |  |  |  |  |
| *Equisetum sp* | 31 | 2.579 | 1.17-3.33 | 31 | 0.291 | 0.12-0.39 | 57 | 1.598 | 0.005-3.6 | 30 | 2.282 | 0.42-8.74 |
| **Sedges** |  |  |  |  |  |  |  |  |  |  |  |  |
| *Carex sp* | 59 | 2.222 | 0.98-3.35 | 59 | 0.216 | 0.10-0.39 | 94 | 0.720 | 0.003-3.62 | 46 | 2.242 | 0.66-4.47 |
| *Eriophorum sp* | 0 | - | - | 0 | - | - | 1 | 0.091 | - | 0 | - | - |
| **Shrub** |  |  |  |  |  |  |  |  |  |  |  |  |
| *Betula nana* | 5 | 2.570 | 1.43-3.49 | 5 | 0.296 | 0.16-040 | 12 | 0.149 | 0.002-1.26 | 2 | 6.186 | 5.14-7.23 |
| *Betula pubescens* | 3 | 2.583 | 1.91-3.20 | 3 | 0.319 | 0.22-0.40 | 5 | 0.365 | 0.003-0.16 | 3 | 4.182 | 3.58-4.80 |
| *Chamaepericlymenum suecica* | 3 | 2.455 | 1.88-2.92 | 3 | 0.251 | 0.19-0.32 | 7 | 0.077 | 0.007-0.16 | 3 | 11.851 | 11.35-12.42 |
| *Empetrum nigrum* | 6 | 0.964 | 0.60-1.31 | 6 | 0.130 | 0.10-0.16 | 22 | 0.035 | 0.005-0.22 | 3 | 6.078 | 5.82-6.36 |
| *Phylodocce caerulea* | 0 | - | - | 0 | - | - | 3 | 0.169 | 0.008-0.44 | 0 | - | - |
| *Rubus chamaemorus* | 5 | 1.995 | 1.44-2.45 | 5 | 0.171 | 0.05-0.25 | 9 | 0.133 | 0.02-0.38 | 5 | 10.754 | 8.18-13.59 |
| *Salix glauca* | 12 | 2.499 | 1.52-3.78 | 12 | 0.306 | 0.11-0.53 | 25 | 0.287 | 0.003-1.58 | 10 | 4.047 | 1.52-8.10 |
| *Salix herbacea* | 34 | 2.556 | 1.78-4.28 | 34 | 0.305 | 0.20-0.52 | 9 | 0.033 | 0.002-0.14 | 1 | 4.166 | - |
| *Salix lapponum* | 11 | 2.590 | 1.21-3.60 | 11 | 0.316 | 0.15-0.48 | 24 | 0.120 | 0.002-0.88 | 7 | 3.930 | 2.85-4.93 |
| *Salix phylicifolia* | 6 | 2.946 | 1.52-4.10 | 6 | 0.389 | 0.13-0.62 | 10 | 0.083 | 0.007-0.44 | 4 | 10.205 | 1.94-14.80 |
| *Salix sp* | 7 | 2.407 | 1.42-3.74 | 7 | 0.259 | 0.19-0.42 | 3 | 0.102 | 0.007-0.28 | 4 | 6.742 | 2.61-8.89 |
| *Vaccinium myrtillus* | 44 | 2.092 | 1.01-3.08 | 44 | 0.213 | 0.08-0.34 | 14 | 0.050 | 0.002-0.29 | 2 | 6.371 | 6.20-6.54 |
| *Vaccinium uliginosum* | 0 | - | - | 0 | - | - | 2 | 0.021 | 0.004-0.04 | 0 | - | - |
| *Vaccinium vitis-idaea* | 3 | 1.090 | 1.03-1.20 | 3 | 0.110 | 0.10-0.12 | 8 | 0.018 | 0.001-0.09 | 0 | - | - |

**Table 4: Parameter estimates and associated 95% CI from generalized linear mixed models for N, P, Si and Ph concentrations fitted as response variables.**

In addition to the fixed effects presented in the manuscript, we added the habitat, reindeer density, and the interaction between grazing intensity and season fitted as fixed effects. Species and sampling site were fitted as random effects (σ^2^). Observations gives the number of samples included in each model. Statistically significant effects are shown in bold.

|  | **N** | | **P** | | **Si** | | **Ph** | |  |
| --- | --- | --- | --- | --- | --- | --- | --- | --- | --- |
| *Predictors* | *Estimates* | *CI* | *Estimates* | *CI* | *Estimates* | *CI* | *Estimates* | *CI* |  |
| Grazing intensity [Summer pasture] | -0.11 | -0.40 – 0.17 | 0.01 | -0.03 – 0.05 | 0.04 | -0.25 – 0.33 | 0.13 | -0.37 – 0.63 |  |
|  |  |  |  |  |  |  |  |  |  |
| Season [Late season] | **-0.62** | **-0.71 – -0.53** | **-0.08** | **-0.09 – -0.06** | **0.25** | **0.16 – 0.34** | 0.26 | -0.12 – 0.64 |  |
| Reindeer density | -0.02 | -0.05 – 0.01 | 0 | -0.01 – 0.00 | 0 | -0.03 – 0.03 | 0.01 | -0.03 – 0.05 |  |
| Habitat [Heath] | -0.09 | -0.19 – 0.01 | 0 | -0.02 – 0.01 | -0.05 | -0.16 – 0.07 | - | - |  |
| C Temp | 0.24 | **0.08 – 0.39** | 0.01 | -0.01 – 0.03 | -0.12 | -0.33 – 0.09 | -0.06 | -0.46 – 0.34 |  |
| Grazing intensity [Summer pasture] × Season [Late season] | -0.04 | -0.19 – 0.11 | 0 | -0.03 – 0.02 | 0.06 | -0.08 – 0.20 | 0.09 | -0.52 – 0.71 |  |
|  |  |  |  |  |  |  |  |  |  |
| Season [Late season] × C Temp | **-0.29** | **-0.39 – -0.18** | **-0.04** | **-0.05 – -0.02** | -0.05 | -0.17 – 0.07 | -0.34 | -0.86 – 0.18 |  |
| **Random Effects** | | | | | | | | |  |
| σ^2^ Species | 0.323 | | 0.006 | | 0.205 | | 8.194 | |  |
| σ^2^ Sampling sites | 0.029 | | 0.001 | | 0.029 | | 0 | |  |
| σ^2^ Residuals | 0.224 | | 0.005 | | 0.279 | | 2.049 | |  |
| Observations | 948 | | 949 | | 1189 | | 528 | |  |

**Figure 2: Chemical composition of plant functional groups in grasslands and heaths.**

Distribution of N, P and Si are represented as a violin plot overlaid with a boxplot for each plant functional group, both in the grasslands and in the heaths. The notches represent the median, the whiskers 1,5 times the inter-quartile range and outliers are showed as points. The thickness of the box is proportional to the number of samples. The distribution of Ph concentrations was not plotted because no sample was estimated for Ph content in the heaths. Horsetails were not present in the heath. Although there is a discrepancy in the number of samples per habitat (n=1250 in the grasslands while n=276 in the heath), both habitats show similar distributions of chemical elements.

**
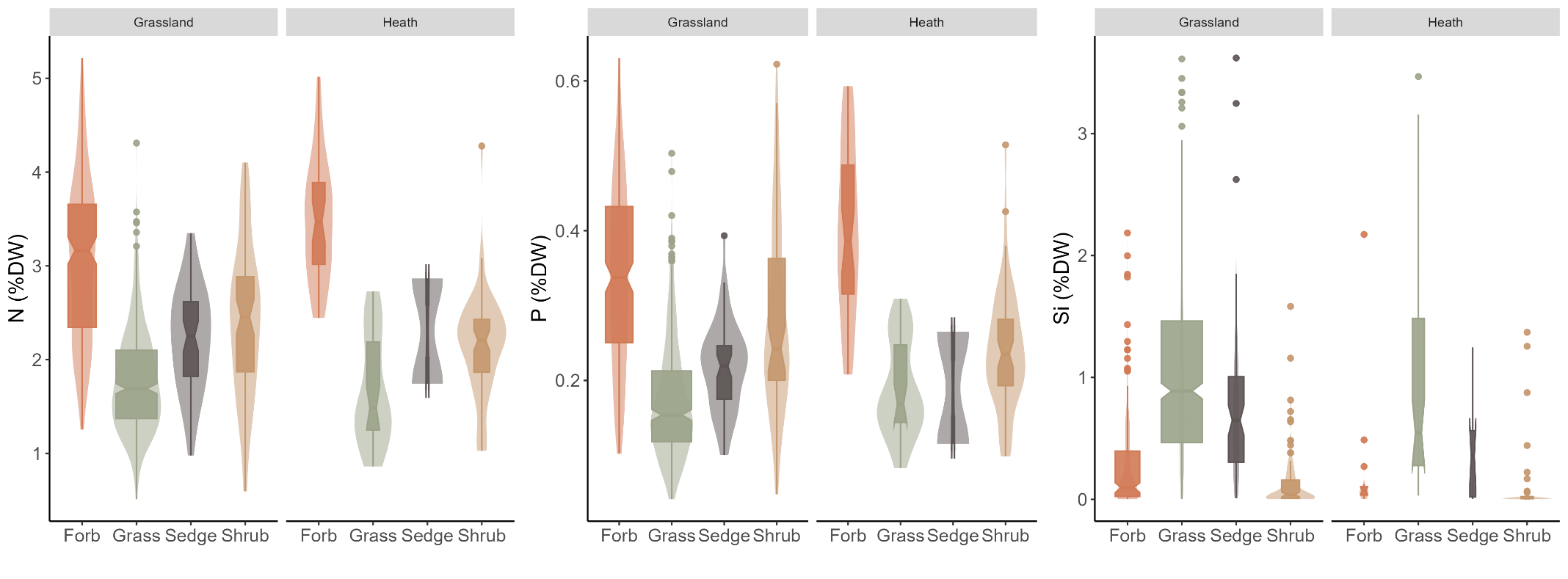
**

**
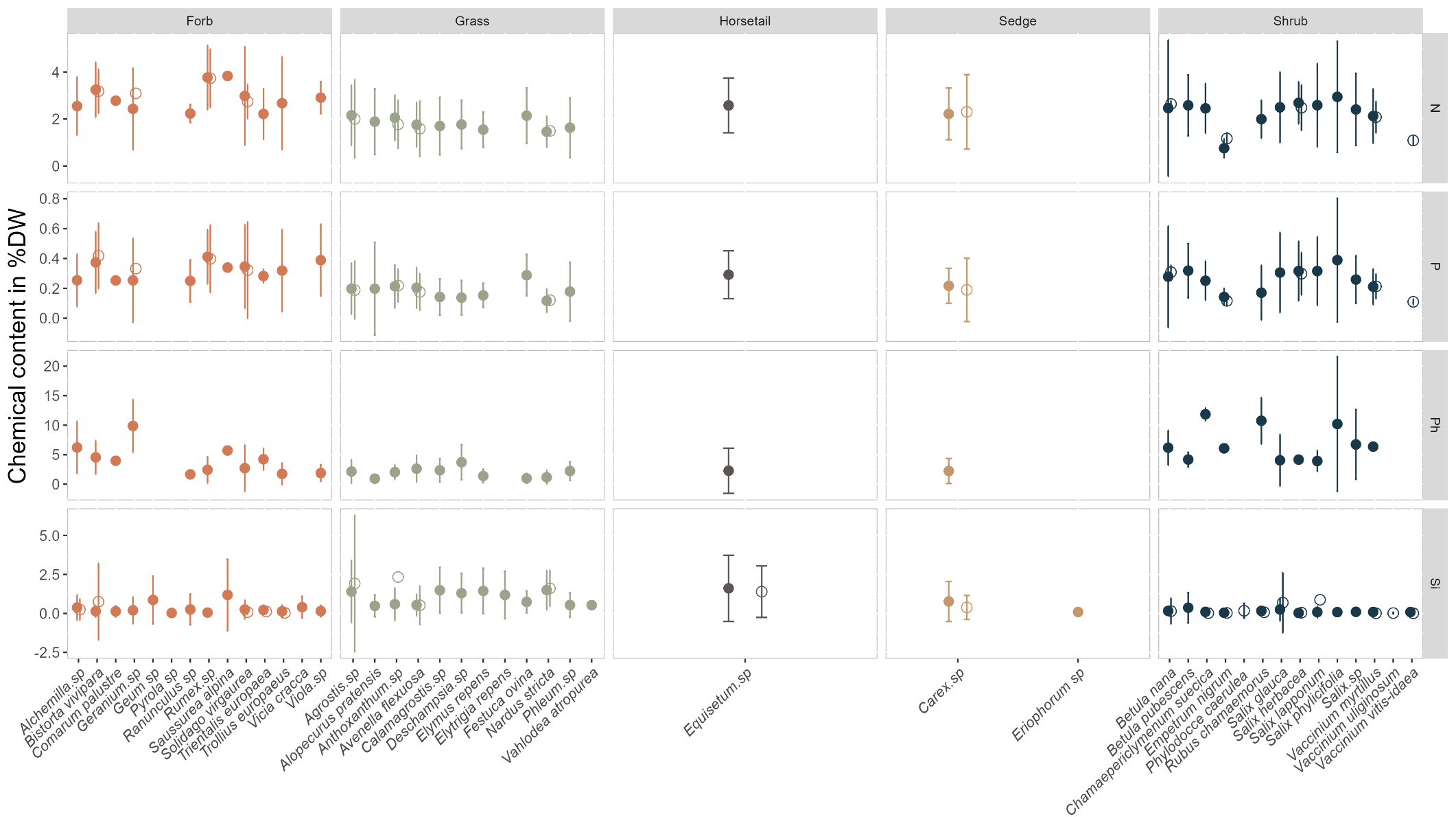
Figure 3: Mean chemical content (N, P, Si and Ph) per species.** The mean chemical content in grasslands (filled circles) and heaths (open circles) was plotted for each chemical compound and for all species (n=43). Error bars represent +/- 2*standard deviation around the mean.


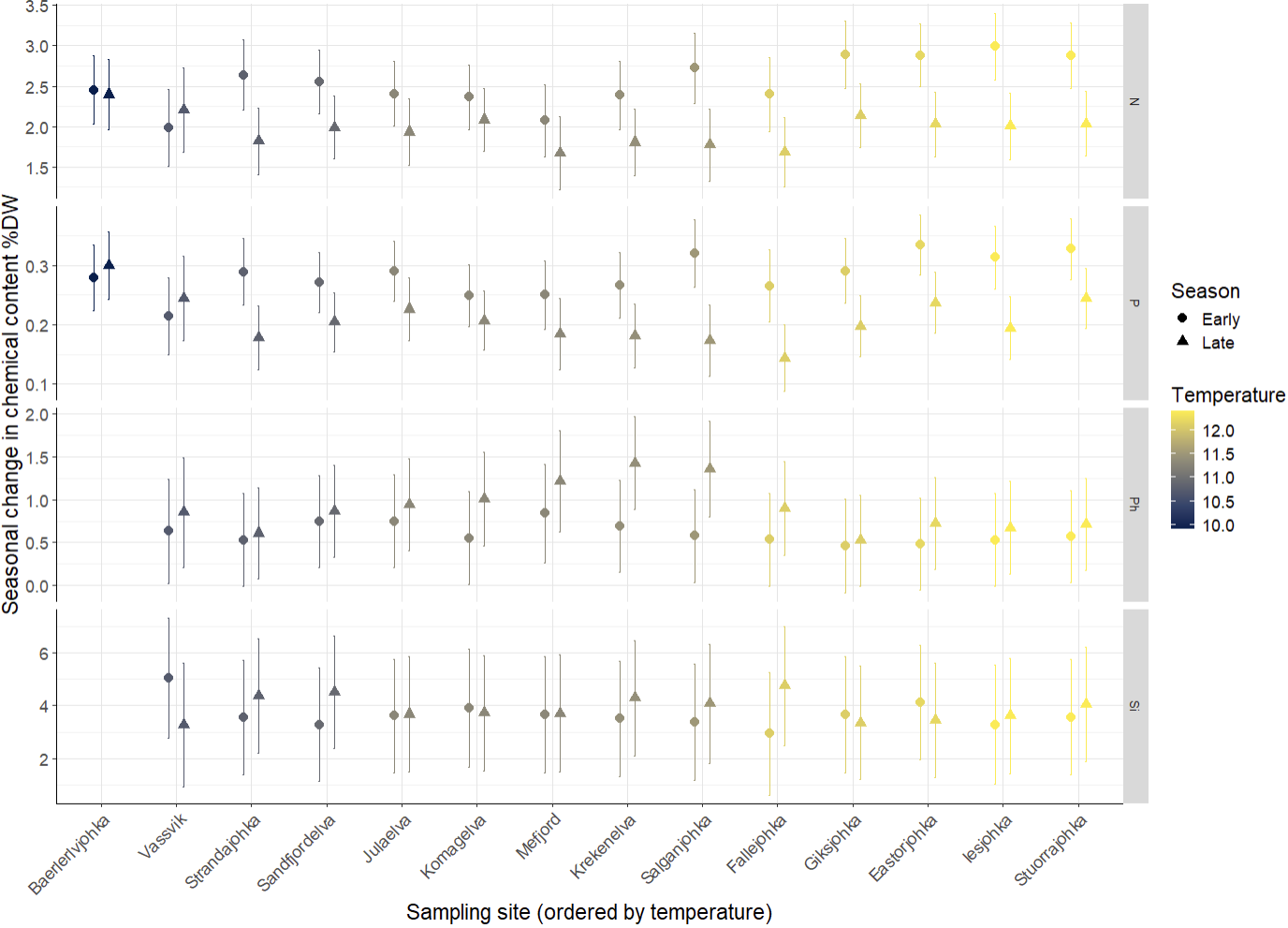
**Figure 4: Average chemical content (N, P, Ph and Si) in both early and late season along a temperature gradient.** Average chemical content was plotted per sampling site. Error bars represent +/- 2 standard error around the mean.

**Table 5: Parameter estimates and associated 95% CI from generalized linear mixed models for N and P concentrations using late season as the reference level for ‘Season’.**

The ‘Season’ fixed effects presented in the manuscript was fitted using ‘late season’ as the reference level. Species and sampling site were fitted as random effects (σ^2^). Observations gives the number of samples included in each model. Statistically significant effects are shown in bold.

The reparameterization shows that the significant effect of C Temp on N for early season samples does not occur for late season samples, highlighting that the steeper seasonal decline in N at warmer sites was mainly due to higher concentrations of N at warmer sites early in the season.

|  | **N** | | **P** | |
| --- | --- | --- | --- | --- |
| *Predictors* | *Estimates* | *CI* | *Estimates* | *CI* |
| Grazing intensity [Summer pasture] | 0.03 | -0.20 – 0.26 | -0.02 | -0.05 – 0.01 |
| Season [Early season] | **0.64** | **0.57 – 0.70** | **0.08** | **0.07 – 0.09** |
| C Temp | -0.05 | -0.21 – 0.11 | **-0.02** | **-0.05 – -0.00** |
| Season [Early season] × C Temp | **0.3** | **0.20 – 0.39** | **0.04** | **0.02 – 0.05** |
| **Random Effects** | | | | |
| σ^2^ Species | 0.330 | | 0.006 | |
| σ^2^ Sampling sites | 0.03 | | 0.001 | |
| σ^2^ Residuals | 0.224 | | 0.005 | |
| Observations | 948 | | 949 | |
